# Three-Step Thermal Drawing for Rapid Prototyping of Highly Customizable Microneedles for Vascular Tissue Insertion

**DOI:** 10.3390/pharmaceutics11030100

**Published:** 2019-02-26

**Authors:** KangJu Lee, Seung Hyun Park, JiYong Lee, Suho Ryu, Chulmin Joo, WonHyoung Ryu

**Affiliations:** Department of Mechanical Engineering, Yonsei University, Yonsei-ro 50, Seoul 03722, Korea; knjulee@gmail.com (K.L.); park.sghn@gmail.com (S.H.P.); jiyonglee@yonsei.ac.kr (J.L.); transcendence81@gmail.com (S.R.); cjoo@yonsei.ac.kr (C.J.)

**Keywords:** microneedle, thermal drawing, polymer bridge, vascular tissue

## Abstract

Microneedles (MNs) have been extensively developed over the last two decades, and highly efficient drug delivery was demonstrated with their minimal invasiveness via a transdermal route. Recently, MNs have not only been applied to the skin but also to other tissues such as blood vessels, scleral tissue, and corneal tissue. In addition, the objective of the MN application has been diversified, ranging from drug delivery to wound closure and biosensing. However, since most MN fabrication methods are expensive and time-consuming, they are inappropriate to prototype MNs for various tissues that have different and complex anatomies. Although several drawing-based techniques have been introduced for rapid MN production, they fabricated MNs with limited shapes, such as thin MNs with wide bases. In this study, we propose a three-step thermal drawing for rapid, prototyping MNs that can have a variety of shapes and can be fabricated on curved surfaces. Based on the temperature control of polymer bridge formation during thermal drawing, the body profile and aspect ratios of MNs were conveniently controlled, and the effect of temperature control on the body profile of MNs was explained. Thermally drawn MNs with different shapes were fabricated both on flat and curved surfaces, and they were characterized in terms of their mechanical properties and insertion into vascular tissue to find an optimal shape for vascular tissue insertion.

## 1. Introduction

The first microneedle (MN) arrays were developed by microfabrication using dry reactive ion etching [1]. This silicon MN array was inserted into human cadaver skin by making microincisions that act as drug delivery channels and increase the permeability of the human skin in vitro by up to four orders of magnitude. Since this first application of MN to human skin, which dramatically improved drug delivery efficiency, MNs have been extensively studied for transdermal drug delivery. As reported by the following studies [2,3,4], the minimally invasive characteristics of MNs were investigated, and the increased permeability afforded by MN has been demonstrated to facilitate the delivery of a wide variety of drugs, from small [5,6] to large molecules [7,8], proteins [9,10], and even genes [11,12]. 

Recently, MNs have been applied not only to the skin but also to other organs, such as blood vessels [13,14,15], eyes [16,17,18,19,20], and so forth. An MN cuff applied to the outside surface of blood vessels as a perivascular drug delivery device to achieve high delivery efficiency to the tunica media has been introduced [13,14]. In addition, perivascular wrappable MN mesh for conformal tight device installation against the pulsatile expansion and constriction of blood vessels has been successfully confirmed [15]. In ocular drug delivery, several MNs have been continuously developed. Laser-cut metal MNs were coated with drugs and manually inserted into human cadaveric sclera [16]. For more advanced localized corneal drug delivery, a MN pen applicator coated with the drug was introduced [17]. Furthermore, with this pen-based platform, a MN was inserted to measure the puncture resistance and fracture toughness of porcine sclera [18]. At the posterior segment of the eye, MNs were shown to be able to deposit drugs into a narrow space between the sclera and the choroid [19]. A tower MN could also deliver drug molecules to the back of the eye for treating retinal disease [20]. 

In addition, the objective of the MN application has been diversified, ranging from drug delivery to wound closure [21] and biosensing [22,23,24,25]. An MN array was inserted between a skin wound, and a biocompatible MN patch was swollen within the tissue and anchored to stick the wound [21]. Hollow MNs [22] and swellable MNs [23] have been used for the extraction of interstitial fluid (ISF) and the detection of the ISF contents. Functionalized MN sensors have been developed as a glucose sensor [24] and a cerebral electrode [25]. In light of this, the range of MN application sites in the biomedical field has been broadened from the skin to the blood vessels, eyes and brain. Since such MNs should be biocompatible, most MNs have been made of a polymer which has low mechanical properties in general. Therefore, it is necessary for overcoming the complex anatomy and different physical properties of each organ to fabricate and find the optimal shape of an MN. 

Since the first MN was developed out of silicon, most MN fabrication methods have been based on conventional microfabrication techniques which can be mainly divided into dry [26,27,28,29,30] and wet etching [31,32,33,34,35]. SF_6_/O_2_ chemicals and the BOSCH process were often combined [27,28] for a high aspect ratio of MN in dry etching. Such a combined dry etching process was further developed to fabricate hollow MNs with a tapered shape [29,30]. On the other hand, wet etching provides a mass production of MNs with lower costs, but control of the shape is more limited than dry etching. For these reasons, drawing lithography has emerged, by which a three-dimensional (3D) polymer structure is directly extended from viscous polymer materials. First, drawing lithography for MN was developed with SU-8 and a precise, machined, stainless micropillar [36]. A spatially discrete, thermal drawing can achieve a specific body profile of MNs using the heating system in both the substrate and the drawing pillar integrated in the micro-z-axis stage [37]. Several types of drawing lithography, such as droplet-born air-blowing drawing [38], electromagnetic drawing [39], magneto-rheological drawing [40], and centrifugal drawing [41] lithography, have been developed in order to produce a larger area MN array or to lift the drug-containing polymer without heating for direct MN fabrication.

These drawing-based approaches can rapidly produce a high aspect ratio (A/R) in MN structures. However, at the same time, this limits the shape of MNs fabricated by the drawing approaches to be only very thin and tall. This often leads to premature bending or collapse of polymeric MNs during their insertion into most tissue barriers. For successful MN insertion into tissues, it is critical to fabricate optimized, custom-shaped MNs for each type of tissue. Most MNs have been fabricated on a flat, two-dimensional (2D) substrate to build MN patches for their applications to skin. Drawing approaches were also limited to the fabrication of MNs on a flat substrate made of the same polymer as the drawn MNs. Thus, more versatile drawing techniques that can fabricate various MN shapes on flat or non-flat surfaces, are necessary to make the approach clinically meaningful in diverse medical applications.

In this study, we propose a three-step thermal drawing technique to rapidly fabricate polymeric MNs that have a wide range of shapes on various surfaces. In particular, it is discussed how temperature differences between a MN substrate and drawing metal pillar can influence the body shapes of thermally drawn MNs. By controlling the thermal drawing parameters, MNs with different A/Rs were fabricated to investigate how the shapes of MNs influenced their insertion into vascular tissue. The structural integrity of thermally drawn MNs with different shapes was compared before and after their insertion into vascular tissue. The shape of the tissue wound was also analyzed using optical coherence tomography (OCT) imaging.

## 2. Materials, Principles and Methods

### 2.1. Materials

The biodegradable polymer, poly(lactic-co-glycolic)acid 90:10 (PLGA 90/10, M_w_ = 268,000 Da, Samyang Biopharmaceuticals, Pangyo, Korea), was used to fabricate an MN array by a three-step thermal drawing. Rhodamine B (product no. R0050, RB) was purchased from Samchun Chemical Inc. (Pyeongtaek, Korea). Stainless steel, micropillar array structures were fabricated by electrical discharge machining (EDM). Each micropillar had a height of 300 µm and a diameter of 260 µm. The pillars were formed in a 3 × 3 array with 700 µm interval. A medical balloon from Fogarty^®^ Occlusion Catheters (620403F, Edwards Lifesciences, Irvine, CA, USA) was used to fabricate MNs on its surface. In order to perform ex vivo insertion tests for MN penetration of vascular tissue with respect to the different A/R of MNs, an abdominal aorta from New Zealand white rabbits (3.0~4.0 kg) was harvested in the operating room.

### 2.2. Thermal Drawing System

**Figure 1:**
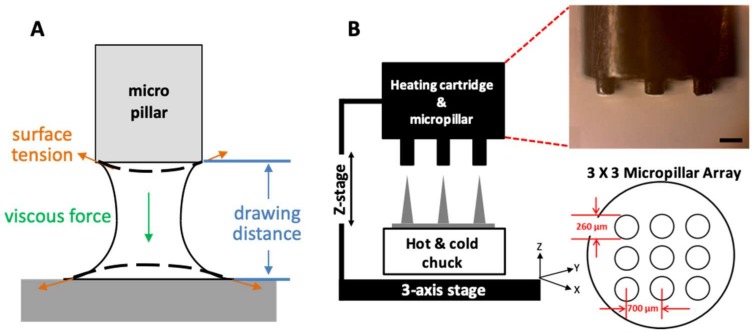
(**A**) A schematic image of phenomenological variables, including surface tension, viscous force, and drawing distance considered in a three-step thermal drawing system. (**B**) A schematic image of three-step thermal drawing system consisting of a hot and cold chuck and heating cartridge for controlling temperature on both sides; hot and cold chuck on the bottom and heating cartridge on the top. A z-axis stage for the precise control of drawing distance and a micropillar array for contacting and drawing polymeric microneedle.

PLGA90/10 film (200 µm thick) made by hot-pressing was used to fabricate an MN array by a three-step thermal drawing system. A custom-built system based on a probe station (Modusys, Hanam, South Korea) was comprised of a hot and cold chuck on a three-axis stage, a precisely machined stainless steel micropillar, and a stereomicroscope (Figure 1A). The temperature of the hot and cold chuck was controlled in a range between room temperature and 250 °C using a temperature controller. Substrate cooling was controlled using a water-cooling system. The substrate was moved manually at a minimum step of 2 µm using a micromanipulator. A micropillar was linked to a z-axis microstage and moved up and down manually. A separate thermal controller was connected to the micropillar to adjust the temperature of the micropillar between room temperature and 260 °C. The 200-µm thick PLGA film on a hot and cold chuck was heated, and then the micropillar array was lowered to make contact with the heated PLGA film. The PLGA was gradually drawn vertically by the micropillar while forming the MN array.

### 2.3. Principle of Three-Step Thermal Drawing for MN

In this study, we focused on the real-time transitional phenomenon of a polymer bridge when the PLGA50/50 film was drawn by a micropillar (Figure 1B). Our three-step thermal drawing system controls the viscosity of the drawn polymer by adjusting the temperature above (micropillar) and below (substrate) the polymer bridge. The drawing distance associated with the MN A/R is also controlled using a micro-z-axis stage, and a sharp MN tip is finally formed by raising the temperature of the micropillar.

**Figure 2:**
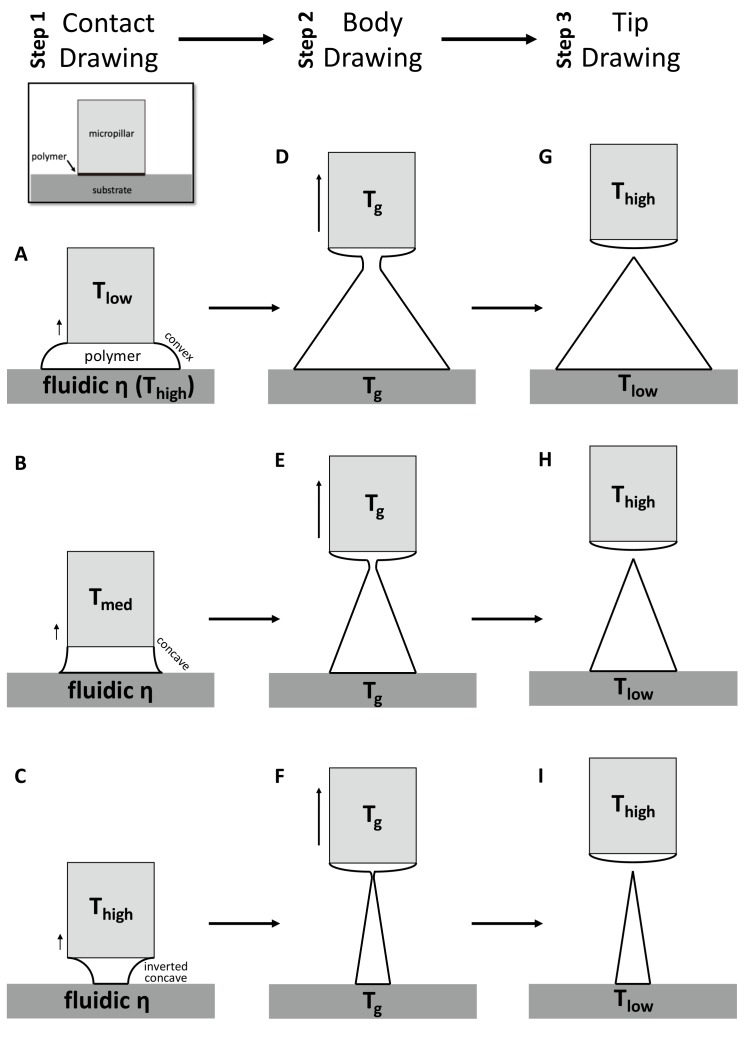
Schematic diagram of three-step thermal drawing process. Contact drawing (**A**–**C**) determinates the aspect ratio (A/R) of microneedle (MN). Body drawing (**D**–**F**) determinates the A/R and height of MN. Tip drawing (**G**–**I**) sharpen the apex tip of MN.

For the first contact drawing, the polymer film is heated to a temperature at which viscosity is sufficiently low (fluidic ƞ). Then, while in contact with the micropillar, the heated polymer substrate is drawn by about 1/10 of the target height (‘Contact Drawing’, Figure 2A–C). When the temperature of the micropillar (*T*_low_) is below the glass transient temperature (*T*_g_) of the polymer, the viscosity of the polymer at the contact surface is relatively high, and the polymer bridge forms a convex shape (Figure 2A). At the midrange of the micropillar temperature (*T*_med_ = *T*_g_), the polymer starts to wet the micropillar surface to a higher degree. This results in the concave profile of the drawn polymer bridge (Figure 2B). When the temperature of the micropillar was increased above *T*_g_ (*T*_high_), the drawn polymer has a low viscosity similar to that of a liquid, and the wetting of the polymer at the surface of the micropillar becomes more significant. This leads to the formation of an inverted concave curvature to the polymer bridge (Figure 2C). In this manner, the side curvature of the drawn polymer is adjusted depending on the micropillar temperature.

Second, the ‘Body Drawing’ determines the height of an MN (Figure 2D–F). Both the temperatures of the substrate and the micropillar (*T*_g_) are equally applied to have uniform viscosity in the drawn polymer bridge. This polymer bridge then has a linear profile while being stretched to the target length. The tip of a MN is sharpened by the final step of ‘Tip Drawing’ (Figure 2G–I). Rapid increase of the tip temperature above a melting temperature increases the mobility of polymer chains. This drives the formation of spherical interface between polymer liquid and the atmosphere at the micropillar. This surface-energy minimizing phenomenon breaks the polymer bridge. Then, the micropillar is immediately retracted and the shape is fixed by lowering the temperature of the polymer MN.

### 2.4. Principle of Transfer Thermal Drawing

**Figure 3:**
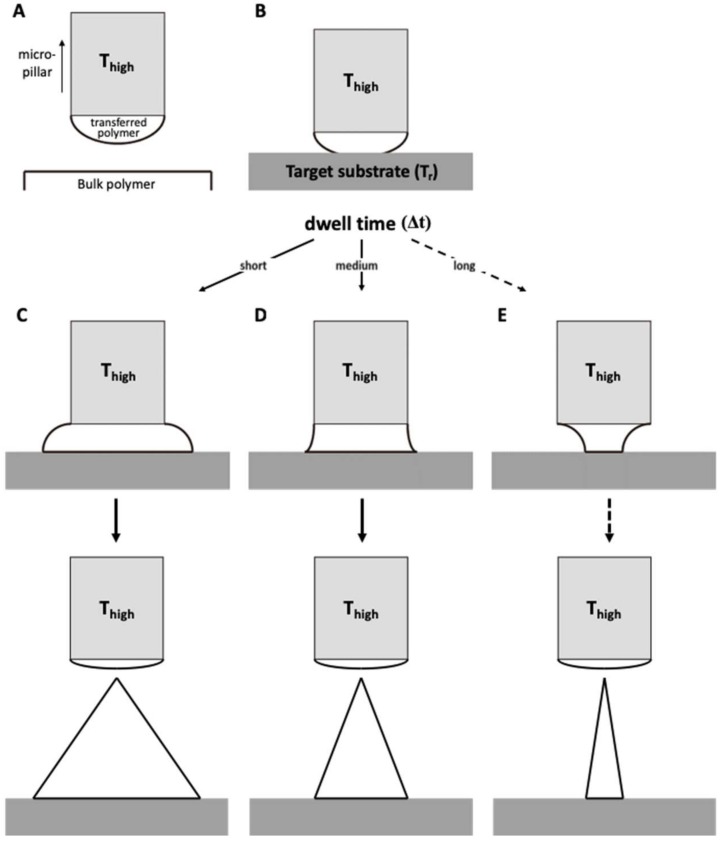
Schematic diagram of (**A**) droplet transfer and (**B**) contact dwelling in transfer thermal drawing process. The shape of the MN via the transfer thermal drawing process can be determined by varying the dwell time to either (**C**) short, (**D**) medium, or (**E**) long.

Transfer thermal drawing can form MNs onto various substrates. First, polymer film is heated on a substrate to form polymer melt. Then, by contact with a heated micropillar, a portion of the polymer melt is transferred from the substrate to the micropillar and forms a droplet of polymer melt (Figure 3A). Unlike the usual three-step thermal drawing, the temperature of a target surface is not controlled (Figure 3B). Thus, to heat the surface sufficiently without additional thermal sources, dwell time (Δ*t*) is typically longer than usual transfer drawing (Figure 3C–E, contact dwelling). In this contact dwelling step, polymer flow is controlled, and the final shape of an MN is determined by adjusting the contact time to the target surface.

In the droplet transfer (Figure 3A), after the contacted polymer is sufficiently fluidized, it is lifted up to form droplets at the tip of the micropillar and moved above a target surface using a 3-axis microstage. After the micropillar is lowered down to the target substrate (Figure 3B), the temperature of the target surface is controlled by adjusting the dwell time (Δ*t*). Depending on the duration of the contact dwelling (short, medium, or long time), the morphology of the polymer bridge between the substrate and the micropillar was controlled like the previous contact drawing of the 3-step thermal drawing. If the Δ*t* is short, the flowability of the polymeric bridge is reduced; then, MNs are formed with a convex curvature, which eventually leads to the formation of bullet-shaped MNs (Figure 3C). Otherwise, a high A/R of MNs with concave curvatures is fabricated for longer Δ*t* (Figure 3D). However, if the target substrate is not stable at high temperatures, it may be difficult to fabricate a slender, high A/R for the MN, as shown in Figure 3E.

### 2.5. Ex Vivo Tissue Insertion Test

MNs with different shapes were prepared using the three-step thermal drawing lithography to find an optimal shape of MNs for their effective insertion into vascular tissue. To achieve successful vascular tissue insertion to a desired depth without structural failures such as bending or buckling, ex vivo insertion tests were performed using MNs with different body profiles. MNs were fabricated to have a height of 650 µm and a tip sharpness of approximately 5 µm with a range of A/Rs varying from 1.5 to 7 (1.5, 2.0, 3.0, 3.5, 4.0, 5.0, 6.0, and 7.0, four MNs each). For the ex vivo insertion tests, a rabbit abdominal aorta was harvested and cut into samples with a width of 20 mm and a length of 5 mm. The prepared tissue samples were then fixed to a customized jig at both ends only, without sagging. Each MN was directed downwards and attached to an auto z-axis. The MN was aligned at the center of the tissue sample and gradually lowered until the MN tip penetrated to a depth of 300 µm from the surface of the vascular tissue at a rate of 10 µm/s. After removal of the MN from the tissue, the vascular tissue samples were fixed for 4 hours in a 10 % formaldehyde solution and dehydrated. The vessel samples were imaged using high-resolution OCT to visualize the MN marks within vascular tissue. The MNs used for the insertion tests were carefully examined via optical microscopy.

### 2.6. Optical Coherence Tomography Analysis

Optical coherence tomography (OCT) imaging was performed at an acquisition speed of 20 fps. After scanning the region of MN insertion, the images were stacked up for three-dimensional (3D) OCT images. Using the 3D OCT images, XZ and YZ cross-sectional images of MN-inserted vascular tissues were obtained from the surface to a depth of 250 μm. Thereafter, the crack sizes formed by MN insertion were estimated by the ImageJ program (NIH). Mechanical deformation of MN against vascular tissue insertion was examined with an optical microscope.

### 2.7. Statistical Analysis

Results were presented as means ± standard error and evaluated by the student *t*-test (2-tailed). *p* < 0.05 was considered to be significant. One-way ANOVA and post hoc Bonferroni tests were used to compare incision depth, incision width, and full width at half depth of a rabbit abdominal aorta with each A/R of the MNs.

## 3. Results

### 3.1. MN Fabrication Using Three-Step Thermal Drawing

**Figure 4:**
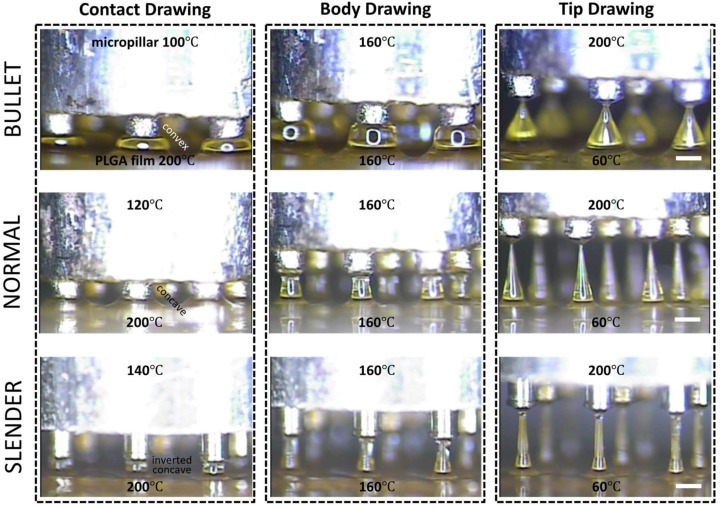
Representative images of shape control for 650 µm height poly(lactic-co-glycolic)acid 90:10 microneedle array with normal (aspect ratio (A/R) = 3.5), bullet (A/R = 1.5), and slender (A/R = 7.0) types with respect to contact, body, and tip drawing via a three-step thermal drawing process (Scale bar = 300 µm).

Three-step thermal drawing for rapid prototyping MNs with various shapes was investigated with the representative three types of MNs as described in Figure 4. Normal, bullet, and slender MNs with the same length of 650 μm were fabricated to have the A/Rs of 3.5, 1.5, and 7, respectively. A PLGA90/10 film was heated up to 200 °C, and a micropillar was heated up to 120, 160, and 200 °C. As shown in Figure 4, when the polymer was drawn to 60 µm height (contact drawing), the body curvature of an MN was adjusted depending on the temperature of the micropillar. After a short pause (5 s for bullet, 15 s for normal, and 40 s for slender shape), the upper (micropillar) and lower (hot and cold chuck) temperatures of the drawn polymer were set at the same 160 °C until uniform viscosity was achieved between the top and bottom of the drawn polymeric bridge. Then, the micropillar was gently lifted up to a drawing length of 650 µm which was the target height of MN (body drawing). Lastly, the temperature of the micropillar was increased to 200 °C to disconnect the polymeric bridge and form the sharp MN tip by inducing droplet formation from the polymer in contact with the micropillar. Immediately after the tip formation, the MN shape was fixed by quenching.

### 3.2. Transfer Thermal Drawing onto Medical Balloon Surface

**Figure 5:**
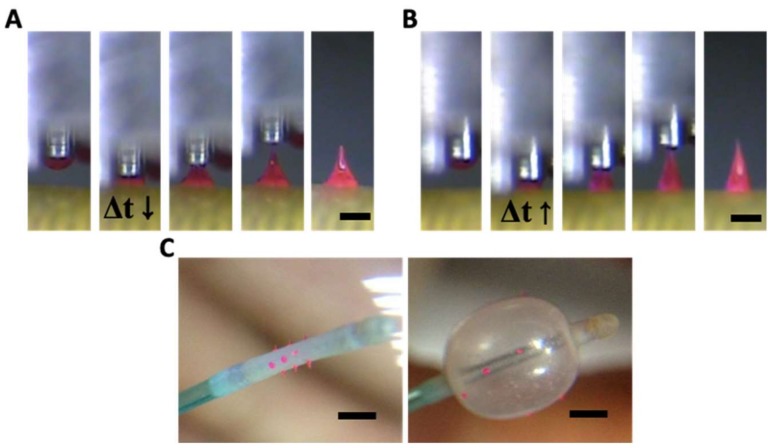
(**A**) Bullet and (**B**) normal shape of poly(lactic-co-glycolic)acid 90:10 microneedle (MN) formed by transfer thermal drawing onto the surface of the medical balloon (Δ*t* indicates dwell time). (**C**) Images before and after MN medical balloon inflated (scale bar = (A,B) 300 µm, (C) 1 mm).

Transfer thermal drawing enables rapid and customized fabrication of MNs directly on a flat or curved surface. In this study, MNs were fabricated on the curved and flexible surface of a medical balloon by the transfer thermal drawing. Bulk PLGA90/10 melt added with 0.1 % (*w*/*w*) rhodamine B for visualization was heated up to 200 °C. A micropillar heated at 200 °C was placed in contact with the bulk PLGA and then drawn up to form a droplet of the polymer melt at the end of the micropillar. This PLGA, droplet-attached micropillar was aligned and placed in contact with the medical balloon surface to make a polymeric bridge between the surface of the balloon and micropillar. The dwell time (Δ*t*) was adjusted to control the curvature of the polymer melt between the micropillar and the balloon surface similarly to the contact drawing step of the three-step thermal drawing. As shown in Figure 5A,B, when the Δ*t* was short (ten s), MNs were formed as a bullet shape (Figure 5A). Otherwise, a normal shape of MN was fabricated as Δ*t* becomes longer (20 s) (Figure 5B). Since the medical balloon was made of elastomer with low thermal conductivity, the polymer bridge between the micropillar and substrate was not sufficiently heated and softened. Thus, even with a longer dwell time than 20 s, a slender MN could not be fabricated. Using a single row of the 3 × 3 array of the micropillar, a 350 µm height of MNs was made on the balloon surface four times at 90º intervals (Figure 5C). MNs on the balloon surface did not fall off of the balloon surface when they were physically pushed or pinched by a tweezer. To confirm the adhesion stability in an aqueous environment, the MN balloon was immersed in water stirred at 350 rpm. After ten minutes, no detachment of MNs from the balloon surface was observed.

### 3.3. Mechanical Behavior of MN During Vascular Tissue Insertion

**Figure 6:**
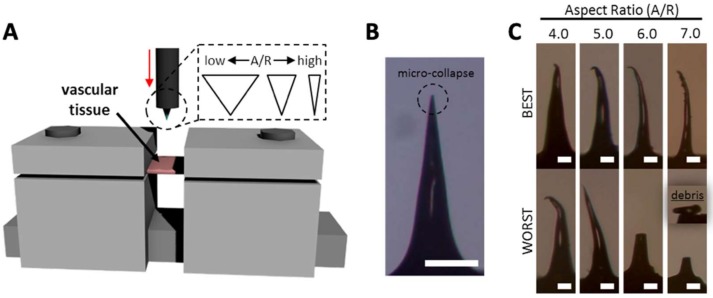
(**A**) A schematic image of ex vivo vascular tissue insertion setup. (**B**) Aspect ratio (A/R) 3.5 microneedle (MN) having micro-collapse at the apex. (**C**) Best and worst case of different A/R of MNs (scale bar = (**B**) 200 µm, (**C**) 50 µm).

Although MNs with high A/Rs are expected to have easier tissue insertion than MNs with low A/Rs, they may be vulnerable to bending or buckling. Therefore, we performed ex vivo tests (Figure 6A) with MNs with various A/Rs to find the optimum MN shape for vascular tissue insertion using a rabbit abdominal aorta. For tissue insertion tests, each MN was inserted into the vascular tissue to a depth of 300 µm from the tissue surface at a speed of 10 µm/s. The images of MNs after ex vivo insertion are shown in Figure 6B, C. MNs with A/Rs from 1.5 to 3.5 were not bent or fractured. In some cases, only the apex of the MN tip micro-collapsed, resulting in a negligible decrease in tip sharpness (from approximately 5 µm to 20 µm) (Figure 6B). Unstable mechanical behaviors of MNs were found in the samples with an A/R of 4.0 and more (Figure 6C). The failure of an MN tip was identified under optical microscopy. In the case of the A/R of 4.0, three of the four samples had only tip bending (the best case is shown in Figure 6C). More severe bending was observed in some sample whose upper body was bent (the worst case). For higher A/Rs, MNs became more vulnerable to bending or fracture during tissue insertion. MNs with an A/R of 6.0 and 7.0 were seriously bent and fractured when the MNs were retracted from the tissue insertion sites. This indicates that MNs with an A/R below 4.0 are suitable for vascular tissue insertion in terms of mechanical stability.

### 3.4. Tissue Wound with Respect to A/R Through OCT Analysis

**Figure 7:**
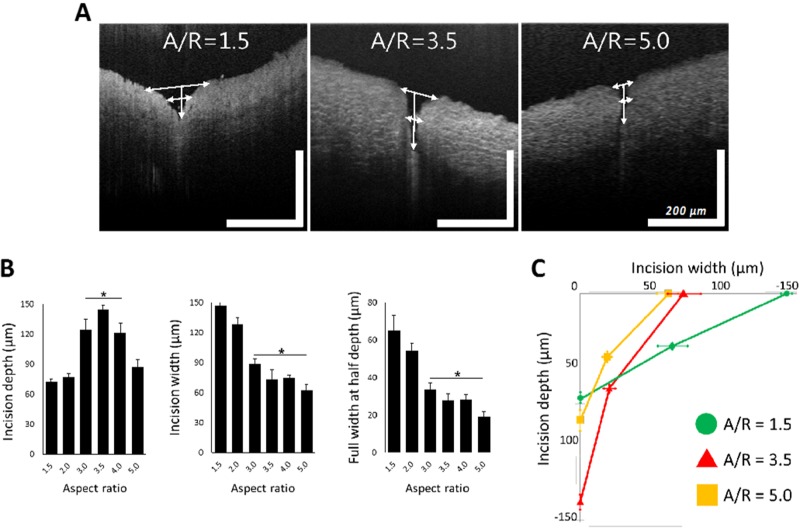
(**A**) Representative optical coherence tomography images of ex vivo inserted tissues with aspect ratio (A/R) 1.5, 3.5, and 5.0 microneedles (MNs). (**B**) Incision depth, incision width, and full width at half depth of rabbit abdominal aorta with different A/R MNs. In the plot of incision depth, * *p* < 0.01, compared with A/R 1.5 and 2.0 group (*p* = 0.002 by ANOVA). In the plots of incision with full width at half depth, * *p* < 0.05, compared with A/R 1.5 and 2.0 group (*p* = 0.007 by ANOVA). All data were presented as the mean ± standard error of the mean. (**C**) Integrated plot from data in Figure 7A, in the case of A/R 1.5, 3.5, and 5.0 MN insertion (scale bar = 200 µm (width and length each)).

MNs with A/R from 1.5 to 5.0 were applied to a rabbit abdominal aorta similarly to the previous ex vivo insertion study. OCT was employed to monitor the shape of the tissue wound caused by MN application. As shown in Figure 7A, the shape of the insertion wounds was different depending on the A/R of MNs. MNs with an A/R of 1.5 did not penetrate deeply, leaving only a relatively large wound area. On the other hand, MNs with an A/R of 5.0 produced a small incision, but they still could not penetrate the tissue to a sufficient depth. In the case of MNs with an A/R of 3.5, the OCT image showed a deep insertion depth and a proper incision area. 

To quantify the geometry of insertion wounds for each MN A/R, the insertion depth, width, and full width at half depth of ex vivo inserted tissues were measured from OCT images (Figure 7B). MNs with A/R ranging from 3.0~4.0 were inserted deeper and resulted in narrower incisions than others. MNs with A/Rs less than 2.0 generated wider incisions without sufficient penetration. High-A/R MNs (A/R = 5) left the smallest incision width, but the insertion depth was insufficient to penetrate the adventitia of a rabbit abdominal aorta—a layer of 100 µm thickness. There was no statistically significant difference among the insertion widths by MNs with A/Rs of 3.0, 3.5, and 4.0. However, since the insertion was the deepest (140 µm) for A/R = 3.5, it can be concluded that an MN with an A/R of 3.5 is optimum for effective vascular tissue insertion.

## 4. Discussion

In this study, we demonstrated rapid prototyping of MNs for vascular drug delivery application. MNs fabricated by a three-step thermal drawing were applied for perivascular drug delivery in previous works [13,14,15]. Smooth muscle cells (SMCs) in the tunica media of the blood vessel can abnormally proliferate and further cause the stenosis or occlusion in the blood vessel. To suppress this, MN should be designed for its insertion into the media layer of blood vessel for perivascular drug delivery. However, the mechanical properties of blood vessels are considered to be viscoelastic [42]. This means that blood vessels exhibit both viscous liquid and an elastic solid [43]. Thus, arteries and veins experience creep and relaxation when placed under a load. In addition, their anatomy is complex, and the tissue has little back support. This makes MN insertion into the vascular tissue much more difficult than their insertion into other tissue such as skin. However, there has been no systematic study of MN insertion into vascular tissue yet. Since the thickness of the adventitia of rabbit abdominal aorta is approximately 100 µm [13,14], perivascular MN should be inserted into the vessel at least up to this depth. We prototyped various shapes of MNs and applied MNs to find which shape of MN is the most suitable for vascular application. It was demonstrated that a PLGA MN with an A/R of 3.5 and 650 µm height was inserted into the middle of the media layer with minimum tissue damage.

In order to apply MNs to various tissues other than the skin, preliminary experiments using various shapes of MNs are required for the effective insertion into each organ or tissue. Research using dry etching for MN fabrication reported 150 µm tall MNs with 45~50 µm diameter [44], 250~300 µm tall MNs with 30~50 µm diameter [29] and 320 µm tall MNs with 10~50 µm diameter [45], which shows that the shape of the MN can be controlled by controlling the ratio of flow rates of the SF_6_ and O_2_ gases used to form the plasma. The wet etching process can also fabricate both cavities (female) and the original (male) shape of an MN. Based on a common process to make trenches in a semiconductor process, a (100) wafer with square mask patterns can be etched with a KOH solution to fabricate pyramidal MN cavities tapered at 54.72º [31]. However, further control of the shape is not possible in this wet etching process even by changing the etchant and adjusting the mask patterning. The fabrication of an embossed MN can be formed with an A/R ranging from 1 to 1.5 by changing various conditions, including chemical etchants, temperature, and mask design [32,33,34]. Though conventional microfabrication technologies are suitable for highly reproducible mass production of MNs, it is time-consuming and expensive to change MN designs and not ideal for MN development stages.

Drawing lithography can also be a solution for rapid prototyping of MNs since it takes less than a few minutes to fabricate the MNs. However, MN shapes that can be fabricated by the method are still limited. Ultra-high A/R MNs were produced to have a length of up to 2 mm and over 100 A/R [36]. Droplet-born air blowing was developed to fabricate different heights of MNs by varying the amount of droplets to be drawn, but the A/R could not be controlled [38]. MNs with the height of 250~480 µm and the very limited A/R of 0.8~1.1 have been made using electromagnetic drawing [39]. Magneto-rheological drawing fabricated MNs with heights of 300–800 µm but with limited A/R [40]. Centrifugal lithography produced MNs with heights of 200~1000 µm but it could not control the A/R either [41].

To control the MN shape during three-step drawing, we utilized temperature-dependent polymeric behavior. As shown in Figure 1B, the polymer needs to be heated to have sufficient mobility of chains to be drawn after contact with a micropillar. Depending on the temperatures of a micropillar and substrate (temperatures at boundaries of the drawn polymer), local points in a polymer bridge have different temperatures. This leads to different polymer chain mobility at each local point of the drawn polymer. When other conditions are maintained identically, the body profile of the polymer bridge is determined by the local mobility of the polymer chains. A portion of the polymer bridge with high chain mobility forms a large concave curvature because of surface tension, while the relatively flat profile forms at the portion with low polymer chain mobility [46,47]. This means that surface tension becomes dominant, and a large curvature can form where polymer chains have high mobility, or at the portions heated at high temperatures. In an extreme case, maintaining a high temperature throughout the entire polymer bridge may break the polymer bridge due to surface tension-driven, large curvature formation [48,49,50]. Based on these characteristics, we controlled MN shapes by applying different temperatures to the bottom (contact drawing), the body (body drawing), and the tip (tip drawing).

Although there is a range of MN shapes that other techniques can produce, the MN shapes and the range of A/R were generally limited. In addition, there have been few guidelines about how to control MN shapes in their fabrication process. In light of this, three-step thermal drawing is simpler to set up than other fabrication methods described above, and MNs can be rapidly prototyped with various shapes and A/Rs ranging from 1.5 to 7.0. Furthermore, transfer three-step thermal drawing still controls the shape of MNs and allows simultaneously polymeric MNs to be fabricated on curved and flexible surfaces like medical balloons, which would be able to enhance the applicability of MN for biomedical usage in the future.

## 5. Conclusions

In this study, we introduced three-step thermal drawing for rapid prototyping MNs for vascular tissue insertion. The principles of the three-step thermal drawing were described. We demonstrated fabrication of MNs with an A/R ranging from 1.5 to 7.0 within a few minutes. In addition, MNs were also fabricated onto a curved and flexible balloon surface for vascular drug delivery. MNs fabricated by the three-step thermal drawing were applied to vascular tissue for perivascular drug delivery. The shape of biodegradable MNs was optimized to insert into a rabbit aorta by mechanical insertion tests and optical coherence tomography analysis.
